# Cohort profile: the Resilient Minds national study of mental health and cognitive resilience in community dwelling adults aged 18 to 93

**DOI:** 10.3389/fdgth.2026.1710349

**Published:** 2026-03-02

**Authors:** Kaarin J. Anstey, Brooke Brady, Lidan Zheng, Jana Koch, Md Hamidul Huque, Michelle K. Lupton, Ralph Martins, Daniel Ashworth, Erin Goddard, Nikki-Anne Wilson, Claudia M. Hillenbrand, Ralf B. Loeffler, Maria Markoulli, Arun V. Krishnan, Tanya Layton, Ranmalee Eramudugolla

**Affiliations:** 1 School of Psychology, University of New South Wales, Sydney, NSW, Australia; 2 Neuroscience Research Australia (NeuRA), Sydney, NSW, Australia; 3 UNSW Ageing Futures Institute, Sydney, NSW, Australia; 4 Brain and Mental Health Program, QIMR Berghofer Medical Research Institute, Herston, QLD, Australia; 5 School of Medical and Health Sciences, Edith Cowan University, Joondalup, WA, Australia; 6 Research Imaging NSW, Prince of Wales Hospital, Randwick, NSW, Australia; 7 School of Optometry and Vision Science, University of New South Wales, Sydney, NSW, Australia; 8 School of Clinical Medicine, University of New South Wales, Sydney, NSW, Australia; 9 Department of Neurology, Prince of Wales Hospital, Sydney, NSW, Australia

**Keywords:** aging, biomarkers, cognition, cohort studies, genetics, resilience, time factors

## Abstract

**Purpose:**

The Resilient Minds (ReMind) cohort was established to investigate cognitive and mental health resilience across the life course, addressing a gap in longitudinal evidence about resilience. The study collected data on traditional medical and lifestyle risk factors for chronic disease, genetics, and a range of mental health and cognitive outcomes. It also aimed to explore contemporary contextual influences on resilience, including internet use, social engagement, environmental exposures, and life course adversities such as perceived discrimination.

**Participants:**

The cohort included 1,640 adults aged 18–93 years, recruited through social media and community groups, to participate in a fully remote, two-year health study. Participants completed online surveys, cognitive and sensory testing, and intensive “sprints” occurring approximately every three months, during which daily surveys and digital health data were collected. A brain-health substudy (BHS) is being conducted for participants aged 50 years and older (current *n* = 184/400 planned), involving to evaluate neuroimaging, blood and ocular biomarkers to assess resilience and cognitive decline.

**Findings to date:**

Thirty percent of participants were born overseas, and the average years of education were 14.7, 15.0 and 14.1 for young, middle aged and older adults, respectively. Among adults aged 65 years and older, 41.9% reported hypertension, 39.1% high cholesterol, 7.1% diabetes, and 22.4% obesity. In the BHS, 18% met criteria for Subjective Cognitive Decline, and 15% met criteria for Mild Cognitive Impairment.

**Future plans:**

The initial study duration is 2 years, with plans to seek funding for extended follow-up to identify long-term predictors of cognitive and mental health resilience and the development of cognitive impairment in ageing.

## Introduction

Within the field of cognitive aging and dementia epidemiology, many studies have been established to specifically examine risk factors for cognitive decline and dementia (1–4). However, few have specifically examined cognitive maintenance or improvement in the face of risk factors in samples that span the adult life course. Understanding the phenomenon of “cognitive resilience” is important because it may shed light on how to prevent cognitive decline. Evaluation of resilience across the whole period of adulthood is important because it allows for the evaluation of the influence of lifestyle and experiences at different ages as a potential determinants of cognitive resilience in aging. Similarly, although there has been a large amount of research on mental health resilience in childhood e.g., (5), there is less research focusing on mental health resilience over the whole adult life course. Longitudinal studies of cognitive or mental health resilience that cover the adult life-course are important because they provide information on within-person changes.

Importantly, there are strong interlinkages between cognition and mental health with common neural substrates and risk factors for their decline, which justifies studying their resilience jointly (6). Prior neuroimaging studies of super-agers have found that they have similar amounts of amyloid (7) but show selective preservation of some functional brain networks, including aspects of the default mode network (8). A better understanding of resilience (9, 10) is crucial for developing interventions to promote health, prevent disease and inform novel precision population health approaches (11–13). The current ReMind study was therefore established to fill this gap in evidence and understanding of resilience. A second aim of the study was to better understand daily experiences using technology, and emerging human activities and experiences that may influence cognitive and mental health outcomes, which have not traditionally been included in cohort studies. These focus on the use of technology, social space and life space.

Although numerous definitions of domain-specific resilience exist in the literature on mental health (14), cognitive aging (11) and physical ageing (15), there is no general consensus (9, 16) on the definition or measurement of resilience. For this study, resilience was broadly defined as better-than-expected functioning (i.e., the expected mean performance) in the face of adversity. Adversity includes both extrinsic and intrinsic factors. Examples of extrinsic factors are life events such as childhood abuse or hardship, job loss, relationship breakdown, experience of violence, discrimination, environmental events such as wildfires, flooding, exposure to trauma, wars and acts of violence, chronic stress, poverty and relocation. Examples of intrinsic factors include genetic risk factors for cognitive and mental disorders or decline, medical conditions including neurological, mental disorders and biological aging. An example operationalisation of cognitive resilience is the demonstration of less memory decline than the expected mean for adults who possess the *APOE e4* allele within a population sample which also includes adults without the *APOE e4* allele (17). Adults with *APOE e4* are at greater risk of Alzheimer's disease.

To explore biological contributors to cognitive resilience, a genetic sub-study was also established to enable the evaluation of genetic predictors of resilience and the association of these with established genetic risk scores for Alzheimer's disease and other dementias. Additionally, to enable more in-depth evaluation of cognitive resilience, a Brain Health Sub-study (BHS) was established to collect neuroimaging, blood, and ocular biomarkers, as well as cognitive assessments for subjective cognitive decline (SCD) and mild cognitive impairment (MCI). The aim of the BHS is to link resilience factors to neural correlates and biomarkers of neurodegenerative disease.

## Cohort description

**Figure 1:**
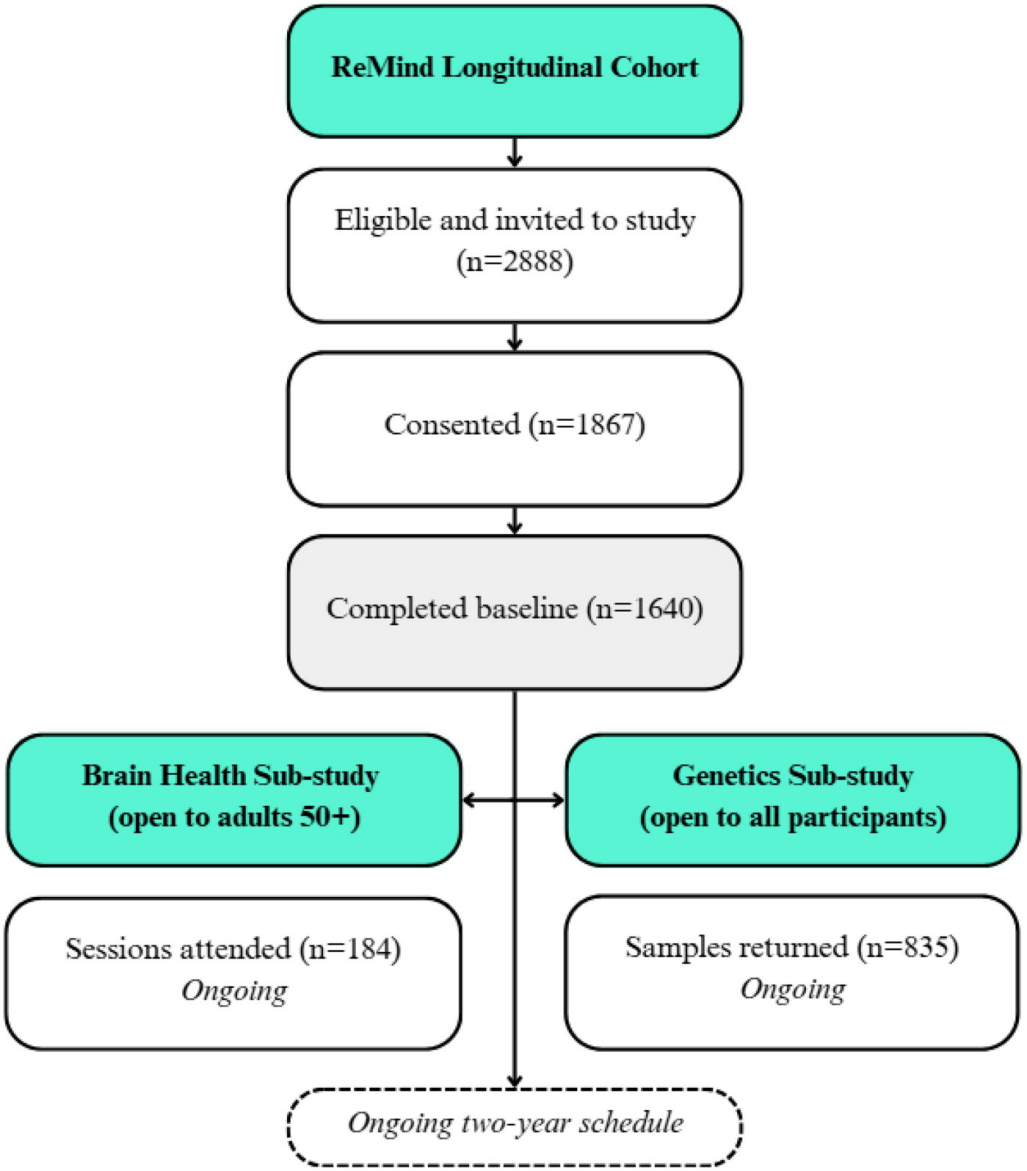
Schematic overview of the resilient minds project, including the resilient minds longitudinal cohort, the brain health sub-study and the genetics sub-study.

The cohort flowchart from recruitment to sub-study selection is shown in Figure 1. Enrolment in the ReMind Longitudinal Cohort occurred between August 2023 and October 2024. The original aim was to recruit 1,600 adults aged 18–95, with approximately 200 participants in each decade (20s, 30s, 40s, 50s, 60s, 70s, 80s, 90s) who would consent to being part of the study for 2 years. Due to logistical constraints, the final sample age distribution varied slightly from the original plan. Participants were recruited from social media platforms, community groups, and the University of New South Wales (UNSW) psychology participant register. Eligible participants were living in Australia, aged 18 or older, proficient in English, iPhone users willing to use their iPhone (model 6S or newer, supporting iOS 13 or later) for the project, with access to Wi-Fi, and willing to wear an Apple Watch (own device or provided by the study). Opt-in invitations for the ReMind genetic sub-study were sent to participants from October 2024. Consenting participants provided a saliva sample for genetic analysis using a mailout saliva collection kit. ReMind participants aged 50 years and older were also invited to participate in the BHS conducted in Sydney, including blood collection, vision assessment, tear sample collection, corneal nerve evaluation, cognitive testing, and brain MRI, which takes approximately 5 h over 1 or 2 sessions. Enrolment in the BHS began in May 2024 and is ongoing with the aim of including 400 participants in the study. Eligible participants are willing to have an MRI, provide a blood sample, provide details of an informant, and undergo eye assessments. Additional participants will be recruited to the ReMind Brain Health Study (who were not originally recruited into the ReMind Cohort) to reach a sample size sufficient for subgroup analysis focusing on SCD.

## Study app

**Figure 2:**
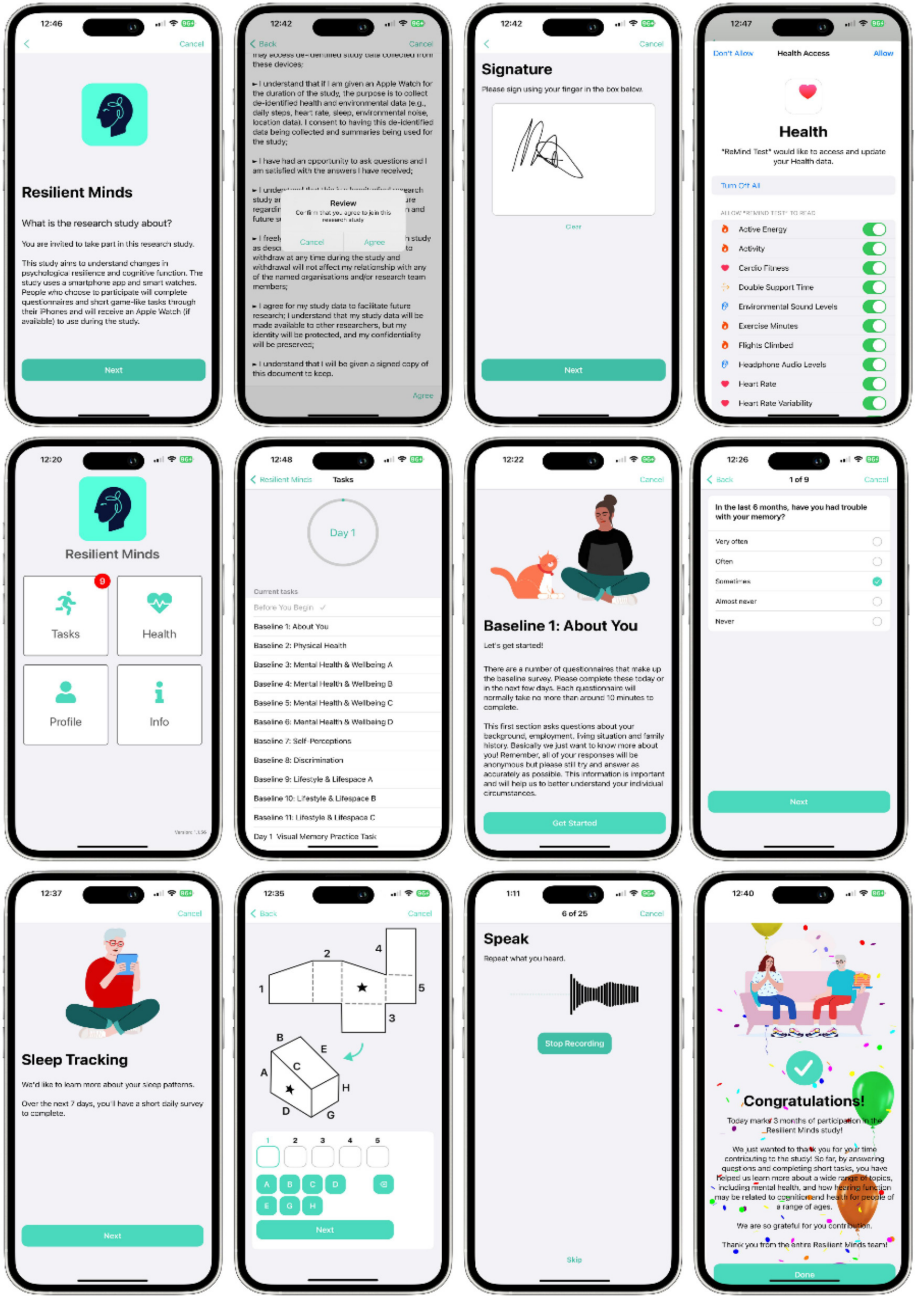
Screen shots from the ReMind app.

ReMind Cohort data is collected using a custom-built Resilient Minds app (Figure 2) for iOS, an extension of an earlier pilot app developed by this team (18). Apple's ResearchKit (https://www.researchkit.org) is used to provide functionality including e-consent, surveys, and game-like cognitive and sensory tasks. The app uses read-only access to the local health store on the participant's iPhone via Apple's HealthKit interface, subject to participant consent. Data is collected continuously throughout the study period from both a worn Apple Watch (if available) and the participant's iPhone.

**Figure 3:**
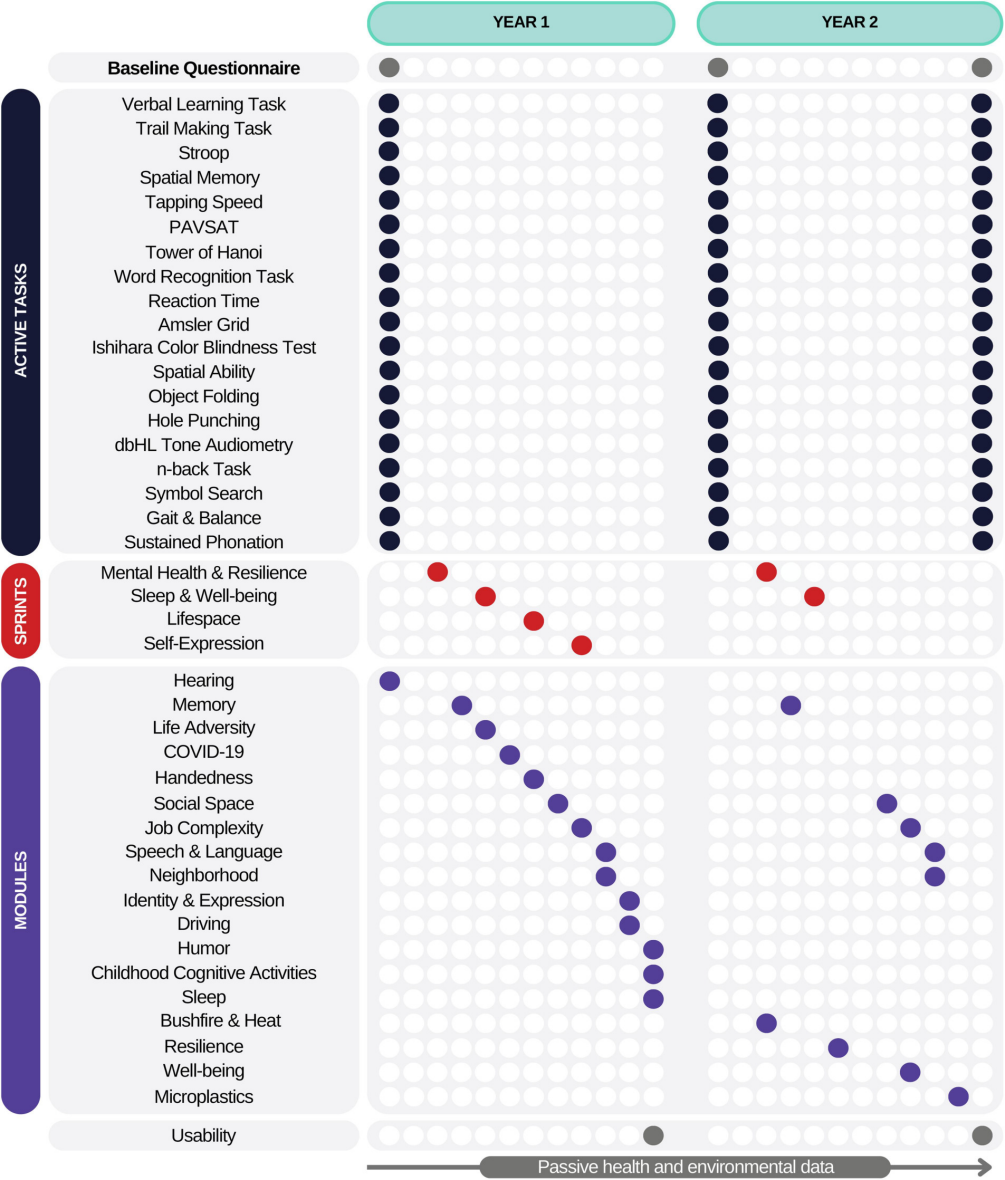
24 month schedule for the resilient minds cohort study.

Figure 3 shows part of the ReMind Cohort schedule administered via the app. Participants complete a comprehensive four-hour baseline assessment (Table 1) within the first two weeks. To track changes over time, key characteristics are re-assessed annually. In-depth, topic-specific data are gathered through one-off “modules”, each requiring 10–20 min. Daily fluctuations in key variables are captured by week-long “sprints”. These sprints, lasting no more than ten minutes daily, may include surveys, tasks, or passive sensor data collection.

**Table 1:** Summary of topics included in the ReMind app surveys.

Survey	Measures
Baseline (demographics and identity)	Age, sex, gender identity, sexual orientation, education, relationship status, household characteristics and postcode, female reproductive and hormone replacement therapies, ethnicity and cultural heritage, language(s), employment, occupation, income, unpaid work, caring, gender typicality and upbringing, discrimination.
Baseline (mental health and wellbeing)	Anxiety, depression, bipolar disorder, schizophrenia, autism, learning disability, ADHD, suicidality, self-efficacy, resilience, emotion regulation, stress, subjective cognitive performance, expectations regarding ageing, self-perceptions of ageing, future time perspective, purpose in life, discrimination, interpersonal relevancy appraisals.
Baseline (physical health, medical conditions, environmental exposures)	General physical health, height, weight, diabetes, blood pressure, stroke, heart conditions, cholesterol, head injury, cancer, hearing, vision, dementia family history, chemical intolerance, environmental exposures, air quality, pesticide exposure.
Baseline (behaviour and engagement)	Alcohol, smoking, vaping, marijuana, food frequency, time use, digital technology and social media use, internet social participation, social networks, everyday activities, travel, sports, performing arts, visual arts, language learning, self-rated physical activity.
Mental Health Sprint	Anxiety, depression, mood, stress, fatigue/vitality, resilience, social contact, time outdoors.
Sleep and Wellbeing Sprint	Attention, sleep quality, insomnia, stress, fatigue/vitality, caffeine intake, resilience.
Life-space Sprint	Self-reported daily activities, life-space, mood, social contact, time outdoors, fatigue/vitality, depression.
Identity and Experience Sprint	Intersectional characteristics and experiences, discrimination.
Hearing Module	Attitudes toward loss of hearing, hearing loss implications and attitudes, awareness of age-related change, subjective age, ideal age, hearing aid use.
Memory Module	Subjective cognitive performance, SCD Plus criteria.
Life Adversity Module	Companionship, loneliness, cumulative lifetime adversity, life events, coping, discrimination
COVID-19 Module	Experiences, impact, vaccines.
Handedness Module	Preference for left or right in hands, feet, eyes and ears.
Social Space Module	Physical and virtual social spaces, social network size, discretionary time, privacy, social support.
Job Complexity Module	Job information, complexity.
Speech and Language Module	Language experience and proficiency.
Neighbourhood Characteristics Module	Neighbourhood characteristics and safety.
Driving Module	Habits, vehicle characteristics, access to transport, future driving plans.
Humour Module	Humour styles, humour in daily life.
Childhood Activity Module	Lifetime cognitive activities, enriching early life activities, high school experiences, other childhood cognitive activities.
Sleep Module	Sleep consistency, napping, sleep quality, sleep behaviours, light exposure, device usage before bed.
Microplastics	
	Exposure to microplastics through drink and food containers and household items
Usability Module	Ease of use and satisfaction, system usability, other feedback.

## Resilience and mental health measures

The range of mental health and psychological constructs assessed at baseline is shown in Figure 3, with details on the measures included in Supplementary materials. The Connor-Davison Resilience Scale (19) and Resilience Scale for Adults (20) were used to measure trait-based resilience. The Cumulative Lifetime Adversity Measure (21) was adapted for this study so that it could be administered on a mobile phone.

## Cognitive and sensory measures

The sensory and cognitive tasks included in the ReMind study are listed in Table 2. Study tasks were sourced from Apple's open-source ResearchKit framework (https://www.researchkit.org), including the Trail Making Test Part A and B, Spatial Memory Task, Stroop Task, Tower of Hanoi, Tapping Speed, Reaction Time, Paced Audio Visual Serial Addition Test, Amsler Grid, Tone Audiometry Task, Speech in Noise, and Sustained Phonation. We adapted the Sustained Phonation Task to allow for the Scripted Speech Task and Unscripted Speech Task. In a previous study (18), we developed the app-based 16-Plate Ishihara Colour Deficiency Test based on the original flash card and paper-based version (22). Of the above, the Trail Making Tasks A and B, the Stroop Task, tone audiometry and the 16-Plate Ishihara Colour Deficiency Test have been previously validated by our team for app-based life-course research (23).

**Table 2:** Cognitive and sensory tasks administered within the resilient minds research app.

Task name	Duration (minutes)	Construct(s) measured
Trail Making Test A	2	Sequencing, visual search, and processing speed
Trail Making Test B	2	Cognitive flexibility, alternating attention, sequencing, visual search, and processing speed
Spatial Memory Task	2	Visuospatial memory and executive function
Stroop Task	3	Cognitive flexibility and inhibitory control
Tapping Speed	2	Motor function, tapping speed, tapping accuracy, and tapping rhythm
Tower of Hanoi	2	Problem solving
Reaction Time	2	Reaction time
PAVSAT	4	Auditory and visual information processing, mental arithmetic
Object Folding	5	Visuospatial ability
Hole Punch task	5	Visuospatial ability
Word Recognition task	3	Vocabulary, Verbal learning
Verbal Learning Task	35	Memory, Verbal learning
Symbol search	2	Processing speed, pattern matching
N-back	2	Working memory
Tone Audiometry task	6	Hearing ability
Speech in Noise	2	Speech intelligibility with competing noise
Sustained Phonation	1	Vocal characteristics
Scripted Speech	2	Reading and vocal characteristics
Unscripted Speech	2	Vocal characteristics and spontaneous speech
16-Plate Ishihara Colour Deficiency Test	3	Red-green colour deficiency
Amsler Grid	1	Visual problems, such as macular degeneration

Novel app-based cognitive tasks were also developed for the Resilient Minds project. Some were based closely on well-validated pen-and-paper measures (24), including versions of the Object Folding Task (24), Hole Punch Task (24), and others while similar to prior tasks involved development of completely new stimuli and contents (a Word Recognition Task, a Verbal Learning Task, an N-back and a Symbol Search task). A summary of tasks administered in the ReMind study app is provided in Tables 1, 2.

## Passive health and environmental data collection

Participants contribute passive health and environmental data throughout the study via their iPhone and a paired Apple Watch (if available). All data collection is contingent upon explicit on-device consent, which participants can modify at any time through their phone settings. See Supplementary Table 2 for details of Apple Watch collected data. For one week (life-space sprint), the app also requests explicit consent to capture GPS information. Location-based information will be analysed to explore daily distance travelled from home, locations visited, time spent in green space and other location-based metrics that may be related to cognitive and/or mental health resilience.

## Brain health substudy

For the classification of SCD and MCI, participants complete a Telephone Montreal Cognitive Assessment (T-MoCA) administered by a trained research assistant. Participants also self-complete an online survey that includes the Everyday Cognition Scale II (25), the Memory Assessment Clinic Questionnaire (26) and additional questions on subjective cognitive decline aligned with (27) SCD I criteria for SCD and SCD-plus. A close friend or family member of the participant also completed the ECogII and other surveys on cognitive and behavioural changes. An algorithmic approach was then used to compare scores on these measures against criteria for SCD, SCD-plus, and MCI (Petersen/NIA-AA and IWG consensus definitions). Participants who are considered to have probable dementia are offered a referral to their general practitioner. Further clinical case review will be undertaken in 2026.

Blood pathology and biomarkers include measures of inflammation, neurodegeneration, liver function and biomarkers of Alzheimer's disease. Brain imaging is undertaken at Research Imaging NSW at the Prince of Wales Hospital, using a 3 Tesla Siemens MAGNETOM Prisma scanner. The imaging sequences are listed in Table 3, with total time in the scanner around 60 min. Structural brain sequences were adapted from the Alzheimer's Disease Neuroimaging Initiative (ADNI) protocol (28). Functional MRI tasks are also conducted. A vision assessment is conducted and includes the Ocular Surface Disease Index (29), the Dry Eye Questionnaire 5-Item (30), *in-vivo* corneal confocal microscopy (IVCCM) and analysis of tear neuropeptides.

**Table 3:** Data collected during brain health sub-study lab sessions.

Domain	Measures
Blood biomarkers	Glucose, HbA1c, cholesterol, Triglycerides, CRP, HDL, LDL, Creatine, Urea, Phosphate, Urate, Liver function tests, CBIL, Complete blood count, Testosterone, Oestradiol, IGF-1, 25 OH Vitamin D, Neurofilament light protein (NfL), Glial fibrillary acidic protein (GFAP), p-tau217, Abeta40/Abeta42.
Cognitive testing	Metamemory Degree of Confidence test.
Physiology	Blood Pressure, heart rate, height, weight, waist circumference.
Structural MRI	Structural MPRAGE, T2 FLAIR, DTI.
Functional MRI	Resting state, Visual flicker task fMRI, Letter Sternberg task fMRI, Hand-Foot Go-No-Go task fMRI.
Vision Assessment	Medical history, medications, Ocular Pain Assessment Scale, Ocular Surface Disease Index, Visual Acuity left and right (high contrast), Vision Biomicroscopy, vision scan, slit lamp test.

HBA1c, glycated hemoglobin; CRP, c-reactive protein; HDL, high density lipoprotein; LDL, low density lipoprotein; CBIL, conjugated bilirubin; IGF-1, insulin like growth factor 1; OH Vitamin D, 25-hydroxyvitamin D; FLAIR, fluid-attenuated inversion recovery MRI; DTI, diffusion tensor imaging; fMRI, functional MRI.

## Patient and public involvement

The study protocol, app design, recruitment strategy, and study materials were reviewed by community members (referred to as “consumers” in Australia) with feedback integrated prior to launch of the ReMind cohort study. A consumer reference group provides ongoing feedback on study materials, recruitment and retention strategies. Participant engagement and retention are supported by email newsletters and feedback on selected tasks. Phone and technical support are available.

## Findings to date

### Cohort summary

The main ReMind study sample comprises 1,640 participants (72.6% female, mean age 52.1 years, age range 18–93), mean education 14.6 years, education range 0–27 years, 30.6% were born overseas, which is similar to the proportion of Australian adults born overseas, estimated to be 31.4% in 2024, by the Australian Bureau of Statistics (31). Table 4 shows some of the descriptives for young, middle and older age groups in the study. There is a greater proportion of participants assigned female at birth than male at birth, and this difference is largest in the youngest age-group. Based on gender identity reported at baseline, there is also a larger proportion of women than men in each age group. The proportion of non-binary participants is largest in the younger age-group (4.0% compared to 0.7% in middle age and 0% in the older group). The majority of participants completed 12 or more years of education. In the younger age group, 19.9% reported height and weight that were classified as obese, 28.9% of the middle aged and 22.4% of the older aged groups are obese.

**Table 4:** Demographics and selected descriptive statistics for the ReMind cohort according to age-group.

Characteristic	Age 18–39 years (*n* = 496)	Age 40–64 years (*n* = 579)	Age 65 + years (*n* = 565)
Sex*			
Male	121 (24.4%)	157 (27.1%)	168 (29.7%)
Female	373 (75.2%)	421 (72.7%)	397 (70.3%)
Another term	2 (0.4%)	1 (0.2%)	–
Gender*			
Man/Male	129 (26.0%)	158 (27.3%)	168 (29.7%)
Woman/Female	346 (69.8%)	414 (71.5%)	397 (70.3%)
Non-binary	20 (4.0%)	4 (0.7%)	–
I prefer another term	1 (0.2%)	3 (0.5%)	–
Sexual Orientation*			
Heterosexual (straight)	319 (64.3%)	513 (88.6%)	528 (93.5%)
Gay or lesbian	46 (9.3%)	33 (5.5%)	24 (4.3%)
Bisexual	82 (16.5%)	16 (2.8%)	9 (1.6%)
I prefer another term	28 (5.6%)	12 (2.1%)	1 (0.2%)
Don't know	18 (3.6%)	2 (0.3%)	2 (0.4%)
Prefer not to answer	3 (0.6%)	3 (0.5%)	–
Years of Education, M (SD)	14.76 (2.67)	15.02 (3.15)	14.10 (3.28)
Secondary or below (<= year 10)	21 (4.1)	39 (6.8)	92 (16.7)
Higher Secondary (Year 11–12)	111 (21.7)	131 (22.7)	140 (25.2)
Post secondary (Year 13+)	379 (74.2)	407 (70.5)	321 (58.2)
Born in Australia	347 (70.0%)	400 (69.1%)	391 (69.2%)
Mother born in Australia	254 (51.2%)	350 (60.4%)	359 (63.5%)
Father born in Australia	256 (51.6%)	319 (55.1%)	336 (59.5%)
Obesity	95 (19.9%)	165 (28.9%)	125 (22.4%)
High blood pressure	44 (9.0%)	147 (25.5%)	237 (41.9%)
High cholesterol	47 (9.7%)	188 (32.6%)	221 (39.1%)
Diabetes	9 (1.8%)	35 (6.1%)	40 (7.1%)
SF-12 Mental Health, M (SD)*	49.29 (11.65)	46.90 (12.73)	44.86 (13.00)
SF-12 Physical Health, M (SD)*	37.22 (11.16)	43.53 (10.78)	49.60 (8.03)
CD-RISC-2, M (SD)*	4.31 (1.82)	4.99 (1.69)	5.29 (1.57)
RSA Total Score, M (SD)*	3.91 (0.22)	3.88 (0.21)	3.84 (0.21)

Descriptive statistics for frequencies reported to in integers with SDs to one decimal place, descriptives for scales are reported to two decimal places.

* Variables marked with an asterisk have different sample sizes.

Of the 1,640 participants, 835 consented to providing saliva for genetic sub study analysis, and 712 samples have currently been collected. The ReMind genetics sub-cohort has an age range of 18–93, mean age of 56.3 and 72.8% female, mean education is 14.7 years.

The ReMind brain health sub-study (BHS) is ongoing with 184/400 participants having completed neuroimaging (fMRI, structural MRI), blood biomarker collection (AB40/42, GFAP, pTau 217 inflammatory markers, metabolic markers), and ocular assessments including tear neuropeptides and corneal imaging. The ReMind BHS sub-cohort has age range of 50–87, mean age of 68.3 and 67.4% female, mean education is 14.2 years. Among the brain health sub-study participants, 18% have psychometrically classified Subjective Cognitive Decline (i.e., meet SCD-I Working Group criteria for SCD plus) (27) and 15% have Mild Cognitive Impairment (MCI). Future participants recruited to the ReMind BHS will be invited into the larger ReMind cohort and recruitment will close by July 2026.

Retention of the overall sample at 12 months is currently 91.4%, with 608 (91.2%) in those aged under 50 and 891 (91.6%) aged over 50. Noting these studies are ongoing, 26 (3.1%) participants have withdrawn from ReMind genetic sub-cohort compared to 3 participants (1.6%) from ReMind BHS.

## Cohort utilization

The large range of variables measured in ReMind and ReMind BHS will allow for several investigations of resilience in both normal ageing and in people with emerging cognitive decline and cognitive impairment. Some of the indicative planned work is noted below.

### Life history and medical event adversities and cognitive resilience

Cross-sectional and longitudinal analysis of the role of life course adversity on cognitive and mental health resilience is planned. We aim to identify characteristics associated with resilience for those with adverse childhood experiences and multiple disease risk factors. Both questionnaire (19) and strategic measures of resilience will be used. Strategic measures involve identifying participants who perform well despite adversities in specific domains as per our previous work (17).

### Social engagement, technology use and cognition

Future research will explore how the physical and digital environments we interact with on a daily basis can influence cognition, health, and wellbeing, with a specific emphasis on social interactions within these spaces and the range and diversity of technology use and engagement.

### Sensory function, resilience and cognition

The study will allow for investigation of how aspects of visual and auditory function interact with resilience in mental health and cognitive domains, and how this varies across the adult life-course. The association of views on ageing and sensory function will also be examined. The ReMind Brain Health sub-study will also provide opportunities to examine how sensory function is associated with functional and structural brain parameters and blood biomarkers.

### Discrimination and resilience

Future research will examine how anticipated and enacted discrimination relate to mental health and cognitive resilience. We will investigate whether overall discrimination dosage—recent and lifetime—differentially impacts well-being compared to identity-specific discrimination experiences based on age, gender, sexual orientation, disability, body size, religion, skin colour, and location. Additionally, we aim to identify protective factors and resilience mechanisms that buffer against discrimination's harmful effects, potentially informing targeted interventions for marginalised populations.

### Prediction of subjective cognitive decline and mild cognitive impairment

The novel design of the study will allow for evaluation of a wide range of predictors of SCD and MCI, including digital biomarkers, language, cognition, Apple Watch metrics, blood biomarkers and neuroimaging. We aim to develop novel risk prediction models that are sensitive to very subtle cognitive deficits.

## Strengths and limitations

The longitudinal cohort's app-based design enables adaptive, cost-effective, and geographically diverse research, embedding the study into daily life. Multi-modal data collection, novel cognitive assessments and biobanking enhance research depth.

Participants consent for future contact facilitating long-term sub-studies and data linkage.

The study is limited by sample bias due to inclusion of iOS users only, who are predominantly female and digitally literate. The sample is not representative, but covers a wide geographical region across the whole continent of Australia. Participant retention over the long term is uncertain. The study depends on honesty of participants to ensure that they are completing the questionnaires and behavioral tasks in an unsupervised environment which increases opportunities for distraction or obtaining assistance. This is a limitation for remotely conducted studies in general. For cognitive testing, review of response times can indicate abnormal patterns of responding.

## Conclusions

The Resilient Minds (ReMind) project, encompassing the longitudinal cohort study, brain health sub-study, and genetics sub-study, will provide invaluable resources for in-depth, long-term research on mental health and cognitive resilience across the adult lifespan. Capitalizing on the combined expertise of its interdisciplinary research team and utilising the advantages of app-based data collection and remote monitoring, ReMind will generate a comprehensive longitudinal dataset including micro-longitudinal data as well as annual assessments of a core set of measures. This rich dataset will allow for deep exploration of how complex life circumstances influence mental health and cognitive resilience across the lifespan. The project's custom-built ReMind app is designed for continuous adaptation, including responsiveness to emerging national research priorities. Collected data and samples will be accessible for diverse research projects, with ongoing collection of self-reported, behavioral, and clinical data further enhancing the resource's value over time. As the project evolves, we anticipate groundbreaking discoveries and insights that will ultimately contribute to improved health and well-being for Australians.
