# Supplementary material for: Cohort profile: the Resilient Minds national study of mental health and cognitive resilience in community dwelling adults aged 18 to 93
Source: Front Digit Health. 2026 Mar 2;8:1710349. doi: 10.3389/fdgth.2026.1710349 (PMC12989522; doi:10.3389/fdgth.2026.1710349)
Supplement: Supplementary file 1 [file Datasheet1.docx]

Supplementary Table 1. Measures included in the ReMind study

| Construct/  Measure | Name of Source Measure | Number of Items | Baseline, Module, Sprint? | Administered on study day(s) |
| --- | --- | --- | --- | --- |
| Sex recorded at birth | ABS Standard for Sex, Gender, Variations of Sex Characteristics and Sexual Orientation Variables | 1 | Baseline | 1 |
| Gender identity | ABS Standard for Sex, Gender, Variations of Sex Characteristics and Sexual Orientation Variables | 1 | Baseline | 1 |
| Sexual orientation | ABS Standard for Sex, Gender, Variations of Sex Characteristics and Sexual Orientation Variables | 1 | Baseline | 1 |
| Education | From Path | 22 | Baseline | 1 |
| Relationship status | 2021 Census Household Form Reference to 2021 ABS census <https://www.abs.gov.au/system/files/documents/12486ae64f0f0ea2d056ee6aa54adc34/Sample%202021%20Census%20Household%20Form%20%5B1.1MB%5D.pdf> | 1 | Baseline | 1 |
| Household members | Developed for ReMind | 6 | Baseline | 1 |
| Accommodation | From PATH | 1 | Baseline | 1 |
| Postcode | 2021 Census Household Form Reference to 2021 ABS census <https://www.abs.gov.au/system/files/documents/12486ae64f0f0ea2d056ee6aa54adc34/Sample%202021%20Census%20Household%20Form%20%5B1.1MB%5D.pdf> | 1 | Baseline | 1 |
| Female reproductive | From PATH, wording adapted for ReMind | 1 | Baseline | 1 |
| HRT | From PATH, wording adapted for ReMind | 2 | Baseline | 1 |
| Aboriginal/TSI | 2021 Census Household Form Reference to 2021 ABS census <https://www.abs.gov.au/system/files/documents/12486ae64f0f0ea2d056ee6aa54adc34/Sample%202021%20Census%20Household%20Form%20%5B1.1MB%5D.pdf> | 1 | Baseline | 1 |
| Country of birth | 2021 Census Household Form Reference to 2021 ABS census <https://www.abs.gov.au/system/files/documents/12486ae64f0f0ea2d056ee6aa54adc34/Sample%202021%20Census%20Household%20Form%20%5B1.1MB%5D.pdf> | 3 | Baseline | 1 |
| Parent country of birth | Australian Bureau of Statistics. (2019). Australian Standard Classification of Cultural and Ethnic Groups (ASCCEG). ABS. <https://www.abs.gov.au/statistics/classifications/australian-standard-classification-cultural-and-ethnic-groups-ascceg/latest-release>. | 4 | Baseline | 1 |
| Cultural background | 2021 Census Household Form Reference to 2021 ABS census <https://www.abs.gov.au/system/files/documents/12486ae64f0f0ea2d056ee6aa54adc34/Sample%202021%20Census%20Household%20Form%20%5B1.1MB%5D.pdf> | 15 | Baseline | 1 |
| Language | 2021 Census Household Form Reference to 2021 ABS census <https://www.abs.gov.au/system/files/documents/12486ae64f0f0ea2d056ee6aa54adc34/Sample%202021%20Census%20Household%20Form%20%5B1.1MB%5D.pdf> | 1 | Baseline | 1 |
| Employment status | No sources recorded | 1 | Baseline | 1 |
| Income | 2021 Census Household Form Reference to 2021 ABS census <https://www.abs.gov.au/system/files/documents/12486ae64f0f0ea2d056ee6aa54adc34/Sample%202021%20Census%20Household%20Form%20%5B1.1MB%5D.pdf> | 2 | Baseline | 1 |
| Occupation | 2021 Census Household Form Reference to 2021 ABS census <https://www.abs.gov.au/system/files/documents/12486ae64f0f0ea2d056ee6aa54adc34/Sample%202021%20Census%20Household%20Form%20%5B1.1MB%5D.pdf> | 3 | Baseline | 1 |
| Housework | 2021 Census Household Form Reference to 2021 ABS census <https://www.abs.gov.au/system/files/documents/12486ae64f0f0ea2d056ee6aa54adc34/Sample%202021%20Census%20Household%20Form%20%5B1.1MB%5D.pdf> | 2 | Baseline | 1 |
| Children | 2021 Census Household Form Reference to 2021 ABS census <https://www.abs.gov.au/system/files/documents/12486ae64f0f0ea2d056ee6aa54adc34/Sample%202021%20Census%20Household%20Form%20%5B1.1MB%5D.pdf> | 3 | Baseline | 1 |
| Deceased children | From PATH | 1 | Baseline | 1 |
| Caregiving | 2021 Census Household Form Reference to 2021 ABS census <https://www.abs.gov.au/system/files/documents/12486ae64f0f0ea2d056ee6aa54adc34/Sample%202021%20Census%20Household%20Form%20%5B1.1MB%5D.pdf> | 4 | Baseline | 1 |
| Height | CogDrisk: A tool to assess risk factors for dementia | 3 | Baseline | 2 |
| Weight | CogDrisk: A tool to assess risk factors for dementia | 3 | Baseline | 2 |
| Diabetes | AUSDRISK: Australian Type 2 Diabetes Risk Assessment Tool | 2 | Baseline | 2 |
| Blood pressure | CogDrisk: A tool to assess risk factors for dementia | 3 | Baseline | 2 |
| Stroke | CogDrisk: A tool to assess risk factors for dementia | 1 | Baseline | 2 |
| Heart condition | CogDrisk: A tool to assess risk factors for dementia | 6 | Baseline | 2 |
| Cholesterol | CogDrisk: A tool to assess risk factors for dementia | 2 | Baseline | 2 |
| Head injury | CogDrisk: A tool to assess risk factors for dementia | 5 | Baseline | 2 |
| Cancer | From PATH | 9 | Baseline | 2 |
| SF-12v2 | SF-12v2 Health Survey: Short Form-12 Version 2 Health Survey | 12 | Baseline | 2 |
| Hearing | The Australian longitudinal study of ageing | 3 | Baseline | 2 |
| Vision | From PATH | 4 | Baseline | 2 |
| Bipolar | Developed for ReMind | 6 | Baseline | 1 |
| Schizophrenia | Developed for ReMind | 6 | Baseline | 1 |
| Autism | Developed for ReMind | 6 | Baseline | 1 |
| Learning disability | Developed for ReMind | 6 | Baseline | 1 |
| ADHD | Developed for ReMind | 6 | Baseline | 1 |
| ADHD screener | WHO Adult ADHD self-report scale-V1.1 Screener | 18 | Baseline | 1 |
| Suicide | Questions adapted for ReMind from: Australian Bureau of Statistics. (2007). National Survey of Mental Health and Wellbeing: Summary of Results. ABS. https://www.abs.gov.au/statistics/health/mental-health/national-study-mental-health-and-wellbeing/2007. Specific terminology changed based on Phase 1 participant feedback i.e. 'end your life' rather than 'committing suicide' | 12 | Baseline | 1 |
| Dementia history | Developed by Kaarin Anstey - adapted AUSDRISK: Australian Type 2 Diabetes Risk Assessment Tool | 18 | Baseline | 1 |
| Center for Epidemiologic Studies Depression Scale | CES-D Scale: Center for Epidemiologic Studies Depression Scale | 10 | Baseline | 1 |
| Self-Efficacy | General Self-Efficacy Scale A measure of self-efficacy | 8 | Baseline | 1 |
| Resilience Scale for Adults | Resilience Scale for Adults | 33 | Baseline | 1 |
| Center for Epidemiologic Studies Anxiety Scale (CESA) | CESA: Center for Epidemiologic Studies Anxiety Scale. | 20 | Baseline | 1 |
| Cognitive emotion regulation questionnaire | CERQ: Cognitive Emotion Regulation Questionnaire. | 18 | Baseline | 1 |
| Subjective Cognitive Performance (MACQ) | MAC-Q: Memory Complaint Questionnaire | 6 | Baseline | 1 |
| CD-RISC2 | CD-RISC2: Abbreviated Connor-Davidson Resilience Scale | 2 | Baseline | 1 |
| Perceived Stress Scale | Perceived Stress Scale (PSS): Measure of perceived stress | 4 | Baseline | 1 |
| Expectations Regarding Ageing | Expectations Regarding Aging (ERA-12): 12-item survey on aging expectations | 12 | Baseline | 1 |
| Self Perceptions of Ageing | Philadelphia Geriatric Morale Scale (ATOA subscale): Measures attitudes toward aging | 5 | Baseline | 1 |
| Lifecourse | Perceiving Time: A Psychological Investigation with Men and Women, Time Estimation Task. | 1 | Baseline | 1 |
| Expected longevity | Desired Lifetime and End-of-Life Desires Measure | 2 | Baseline | 1 |
| Gender expression | 2-item protocol adapted from: Socially Assigned Gender Nonconformity (SAGN) Measure | 2 | Baseline | 1 |
| Childhood Gender Role Nonconformity | Recalled Childhood Gender Identity/Gender Role Questionnaire (RCGI/R) | 3 | Baseline | 1 |
| Genders in Childhood Home | Developed for ReMind (KJA and BB) | 1 | Baseline | 1 |
| Social Relevancy | Developed for ReMind (BB and LZ). Piloted as part of Social Relevancy study. | 18 | Baseline | 1 |
| Future Time Perspective | Future Time Perspective Scale | 10 | Baseline | 1 |
| Purpose in Life Scale | Midlife Development in the United States (MIDUS II) Psychosocial Constructs and Composite Variables | 7 | Baseline | 1 |
| Discrimination | Intersectional Discrimination Index | 44 | Baseline | 1 |
| Time Use | Developed for ReMind. Used in Social Relevancy Pilot (BB & LZ) | | Baseline | 1 |
| Digital Technology | Sedentary behaviour - Digital technology | 3 | Baseline | 1 |
| Social media | Social media- UK Millennium Cohort Study (MCS) | 3 | Baseline | 1 |
| Internet Social Participation Scale | Social Participation Measure for Older Adults' Internet Use | 6 | Baseline | 1 |
| Social network | Lubben Social Network Scale: Measures social networks in elderly populations | 6 | Baseline | 1 |
| Everyday activities - from CogDrisk | CogDrisk: A tool to assess risk factors for dementia | 17 | Baseline | 1 |
| Travel | Developed for ReMind | 10 | Baseline | 1 |
| Sport | Developed for ReMind | 63 | Baseline | 1 |
| Performing arts | Developed for ReMind | 31 | Baseline | 1 |
| Visual arts | Developed for ReMind | 31 | Baseline | 1 |
| Language | Developed for ReMind | 31 | Baseline | 1 |
| CSIRO Food Frequency Questionnaire (partly used in CogDrisk) | Computerized Dietary Analysis System | 44 | Baseline | 1 |
| Alcohol | Alcohol Use Disorders Identification Test (AUDIT) | 10 | Baseline | 1 |
| Smoking | Smoking and Mental Health Measure- "Adapted from PATH. Original source: Stress and Well Being Project: Jorm AF, Rodgers B, Jacomb PA, Christensen H, Henderson S, Korten AE (1999). Smoking and Mental health: results from a community survey. Medical Journal of Australia, 170: 74-77 | 8 | Baseline | 1 |
| Vape | Vaping Behavior Measure | 2 | Baseline | 1 |
| Marijuana | Developed for ReMind. Adapted from AIHW NDSHS vaping questions <https://www.aihw.gov.au/reports/illicit-use-of-drugs/national-drug-strategy-household-survey/contents/tobacco-and-e-cigarettes-vapes> | 8 | Baseline | 1 |
|  |  |  |  |  |
| IPAQ SF | International Physical Activity Questionnaire (IPAQ) | 5 | Baseline | 1 |
|  |  |  |  |  |
| Chemical Intolerance | Brief Environmental Exposure and Sensitivity Inventory (BREESI). | 3 | Baseline | 1 |
|  |  |  |  |  |
| Environmental Exposure | Kaarin Anstey. *Note question expanded April 2019 in order to collect more information about duration and frequency of exposure, in occupational and domestic environments. Guiding References included: Park, S., Kim, S. K., Kim, J. Y., Lee, K., Choi, J. R., Chang, S. J., . . . Koh, S. B. (2019). Exposure to pesticides and the prevalence of diabetes in a rural population in Korea. Neurotoxicology, 70, 12-18. AND: Everett, C. J., & Matheson, E. M. (2010). Biomarkers of pesticide exposure and diabetes in the 1999-2004 National Health and Nutrition Examination Survey. Environment International, 36(4), 398-401. | 1 | Baseline | 1 |
| Perceptions of Air Quality | Developed for ReMind. | 2 | Baseline | 1 |
| Attitudes toward loss of hearing | Attitudes Toward Loss of Hearing Questionnaire (ALHQ) | 27 | Hearing Module | 22 |
| Hearing loss implications and attitudes |  | 12 | Hearing Module | 22 |
| Awareness of age-related change (AARC) | Awareness of Age-Related Change (AARC) Measure | 10 | Hearing Module | 22 |
| Subjective age and ideal age | Subjective Age and Ideal Age Measure | 2 | Hearing Module | 22 |
| Hearing aids screening question | Developed for ReMind | 2 | Hearing Module | 22 |
| GAD (Generalized Anxiety Disorder) | Generalized Anxiety Disorder 7 (GAD-7) | 2 | Mental Health Sprint | 50 (1-7) |
| Mood | Positive and Negative Affect Schedule (PANAS) | 12 | Mental Health Sprint | 50 (1-7) |
| PHQ-9 (Depression) | Patient Health Questionnaire-9 (PHQ-9) | 9 | Mental Health Sprint | 50 (1-7) |
| Stress | Developed for LWW | 4 | Mental Health Sprint | 50 (1-7) |
| Fatigue | SF-36 health | 4 | Mental Health Sprint | 50 (1-7) |
| CD-RISC (Resilience) | Connor-Davidson Resilience Scale (CD-RISC) | 2 | Mental Health Sprint | 50 (1-7) |
| Social contact | Developed for ReMind | 3 | Mental Health Sprint | 50 (1-7) |
| Time Outside | Developed for ReMind | 1 | Mental Health Sprint | 50 (1-7) |
| Screening question | Developed for ReMind | 1 | Memory Module | 106 |
| Subjective Cognitive Performance | Memory Assessment Clinics Questionnaire (MAC-Q) | 6 | Memory Module | 106 |
| SCD Plus Criteria | Subjective Cognitive Decline Plus (SCD-Plus) Criteria | 6 | Memory Module | 106 |
| Companionship/Loneliness | UCLA Loneliness Scale (Version 3) | 3 | Life Aversity | 127 |
| Cumulative Lifetime Adversity Measure | Cumulative Lifetime Adversity Measure | 51 | Life Aversity | 127 |
| Life Events | List of Threatening Experiences (LTE) | 15 | Life Aversity | 127 |
| Qualitative Coping Question | Developed for ReMind | 1 | Life Aversity | 127 |
| Attention | Modified Adult ADHD Self-Report Scale (ASRS) 6-item SF | 6 | Sleep & Wellbeing | 134 |
| Quantitative Sleep | Developed for ReMind | 2 | Sleep & Wellbeing | 134 |
| Insomnia Severity Index | Insomnia Severity Index (ISI) - Adapted wording for daily testing | 7 | Sleep & Wellbeing | 134 |
| Stress | Developed for ReMind | 4 | Sleep & Wellbeing | 134 |
| Fatigue / Vitality | SF-36 Vitality Subscale | 4 | Sleep & Wellbeing | 134 |
| Caffeine | Computerised Dietary Analysis system | 7 | Sleep & Wellbeing | 134 |
| CD-RISC | Connor-Davidson Resilience Scale | 2 | Sleep & Wellbeing | 134 |
| Questions adapted from Oelsner MESA COVID-19 questionnaire | -Questions adapted from Oelsner MESA COVID-19 questionnaire | 8 | Covid 19 | 162 |
| COVID-19 Impact Questionnaire | Developed for PATH/ReMind | 14 | Covid 19 | 162 |
| Vaccines | Developed for ReMind | 4 | Covid 19 | 162 |
| Edinburgh Handedness Inventory (10-item version) | Edinburgh Handedness Inventory (10-item version) | 10 | Headness | 190 |
| Handedness Inventory (modern items) | Developed for ReMind | 7 | Headness | 190 |
| Footedness, Eyedness, Eardness | Lateral Preference Inventory | 3 | Headness | 190 |
| GPS Measure (iPhone) |  | 2 | Lifespace_Sprint | 217 |
| Self-reported Daily Activities | Adolescent time use and mental health: Behavioural Exposure Data - Time use diaries | 45 | Lifespace_Sprint | 218 |
| Lifespace Questionnaire | Life Space Questionnaire | 10 | Lifespace_Sprint | 218 |
| Mood | 11-Mood Items | 12 | Lifespace_Sprint | 218 |
| Social Contact and Time Outdoors | Ecological Momentary Assessment (EMA) | 4 | Lifespace_Sprint | 218 |
| Fatigue/Vitality | SF-36 Vitality Subscale | 4 | Lifespace_Sprint | 218 |
| PHQ-9 (Depression) | Patient Health Questionnaire-9 | 9 | Lifespace_Sprint | 218 |
| Social space types | Developed for social space pilot study (BB & LZ)/ReMind | 12 | Social Space | 232 |
| Social network size | Developed for social space pilot study /ReMind | 2 | Social Space | 232 |
| Physical spaces | Developed for social space pilot study /ReMind | 2 | Social Space | 232 |
| Virtual spaces | Developed for social space pilot study /ReMind | 1 | Social Space | 232 |
| Friend and family member | Developed for social space pilot study /ReMind | 7 | Social Space | 232 |
| Frequency type | Developed for social space pilot study /ReMind | 12 | Social Space | 232 |
| Time type | Developed for social space pilot study /ReMind | 11 | Social Space | 232 |
| Scenarios | Developed for social space pilot study /ReMind | 3 | Social Space | 232 |
| Discretionary time | Developed for social space pilot study /ReMind | 3 | Social Space | 232 |
| Privacy | Developed for social space pilot study /ReMind | 2 | Social Space | 232 |
| Social support | Developed for social space pilot study /ReMind | 3 | Social Space | 232 |
| Job Details | Developed for ReMind | 5 | Job Complexity | 260 |
| Task Specifications | Dictionary of Occupational Titles (DOT) Index; Also added new domains for creative complexity (not included in DOT) | 30 | Job Complexity | 260 |
| Subjective Impact | Developed for ReMind | 2 | Job Complexity | 260 |
| Speech Therapy | Developed for ReMind | 2 | Speech Language | 274 |
| Sustained phonation | Maximum Phonation time | 3 | Speech Language | 274 |
| Language Sample |  | 1 | Speech Language | 274 |
| Language Experience and Proficiency Questionnaire (LEAP-Q) | Language Experience and Proficiency Questionnaire (LEAP-Q) | 257 | Speech Language | 274 |
| Neighbourhood | Neighborhood Environment Walkability Scale (NEWS-AU) | 31 | Neighbourhood | 288 |
| Neighbourhood New Items | Developed for ReMind | 7 | Neighbourhood | 288 |
| Gender Related Variables | 25-item Gender-related variables for health research measure. | 21 | Identity & Experience | 302 |
| Discrimination | Intersectional (dis)advantage | 53 | Identity & Experience | 302 |
| Gender | Repeated from Baseline | 5 | Identity & Experience | 302 |
| Gender stability | Developed for ReMind | 4 | Identity & Experience | 302 |
| Contexts | Developed for ReMind | 23 | Identity & Experience | 302 |
| Driving habits |  | 3 | Driving Module | 316 |
| Vehicle | Adapted from Anstey driving studies | 5 | Driving Module | 316 |
| Car Safety Features | Features listed are 12 of the most commonly used ADAS features in Australian vehicles as advised by Professor Mike Regan: a CI on the Linkage Cognitive Driving Grant | 12 | Driving Module | 316 |
| Future years |  | 1 | Driving Module | 316 |
| Driving habits |  | 11 | Driving Module | 316 |
| Transport | Adapted from National Ageing Study (AFI) | 30 | Driving Module | 316 |
| Licenses | Developed for ReMind | 2 | Driving Module | 316 |
| Humour Styles Questionnaire | Humor Styles Questionnaire (HSQ) | 32 | Humour | 330 |
| Humour in Daily Life | Developed for ReMind | 18 | Humour | 330 |
| Rush Alzheimer's Disease Centre Lifetime Cognitive Activity Scale | Rush Alzheimer's Disease Centre Lifetime Cognitive Activity Scale | 11 | Childhood Activity | 344 |
| Hopkins - Enriching Early-life Activities | Enriching Early Life Activities (EELAs) | 10 | Childhood Activity | 344 |
| RICE Survey Questions + New Items | Retrospective Indigenous Childhood Enrichment Scale (RICE) | 14 |  |  |
| High School Experiences | Enriching Early Life Activities (EELAs) | 11 | Childhood Activity | 344 |
| Other Childhood Activities | Developed for ReMind | 2 | Childhood Activity | 344 |
| Sleep Consistency | Developed for ReMind | 1 | Sleep | 359 |
| Napping | 6 Items Napping Behaviour | 9 | Sleep | 359 |
| SLEEP -50 | SLEEP-50 Questionnaire | 43 | Sleep | 359 |
| Lighting | Developed for ReMind. Based on evidence: Carter B, Rees P, Hale L, Bhattacharjee D, Paradkar MS. Association Between Portable Screen-Based Media Device Access or Use and Sleep Outcomes: A Systematic Review and Meta-analysis. JAMA Pediatr. 2016 Dec 1;170(12):1202-1208. doi: 10.1001/jamapediatrics.2016.2341. PMID: 27802500; PMCID: PMC5380441. | 2 | Sleep | 359 |
| SLEEP -50 | SLEEP-50 Questionnaire | 7 | Sleep | 359 |
| REM Sleep Behaviour Disorder Questionnaire | REM Sleep Behaviour Disorder Questionnaire | 14 | Sleep | 359 |
| Electronic device usage | Sleep Related Questionnaire | 3 | Sleep | 359 |
| General Usability | Developed for ReMind | 4 | Usability | 365 |
| mHealth app usability questionnaire (including ease of use and satisfaction subscale) | mHealth app usability questionnaire (including ease of use and satisfaction subscale) | 8 | Usability | 366 |
| Post-Study System Usability Questionnaire (PSSUQ) - Modified by replacing word ësystemí with ëresearch appí. | Post-Study System Usability Questionnaire (PSSUQ) | 18 | Usability | 367 |
| Feedback | Developed for ReMind | 4 | Usability | 368 |
| Parkinson’s Disease Risk Factor Questionnaire (PD-RFQ) | Parkinson’s Disease Risk Factor Questionnaire (PD-RFQ) | 21 | Baseline 2 | 366 |
| PESTICIDE | Parkinson’s Disease Risk Factor Questionnaire (PD-RFQ) | 1103 | Baseline 2 | 366 |

**Supplementary Table 2.**

ReMind study measures collected from Apple watch

| Table 3. Daily passive health and environmental information collected using the iPhone and/or Apple Watch (if available). | | |
| --- | --- | --- |
| Measure | Description | Device |
| Watch wear hours | Number of hours that Apple Watch was worn during the day. | Apple Watch |
| Active kilojoules | Aggregate active energy used. | Apple Watch |
| Basal kilojoules | Estimated aggregate basal energy used. | Apple Watch |
| Step count | Number of steps taken. | Apple Watch |
| Exercise minutes | Number of minutes of exercise. | Apple Watch |
| Walk/run kilometres | Number of kilometres covered by walking or running. | Apple Watch |
| Flights climbed | Number of flights of stairs climbed. | Apple Watch |
| Stand time (minutes) | Number of minutes spent standing. | Apple Watch |
| Stand time (hours in day) | Number of hours in which the participant was standing for at least a minute. | Apple Watch |
| Vo2 max | Estimate of maximum oxygen consumption during exercise. | Apple Watch |
| Sleep hours | Hours:mins of sleep. | Apple Watch |
| Sleep time (REM) | Hours:mins of Rapid Eye Movement (REM) sleep. | Apple Watch |
| Sleet time (core) | Hours:mins of core sleep. | Apple Watch |
| Sleep time (deep) | Hours:mins of deep sleep. | Apple Watch |
| Sleep time (unspecified) | Hours:mins of sleep with type unspecified | Apple Watch |
| Average resting heart rate | Average resting heart rate. | Apple Watch |
| Average walking heart rate | Average heart rate when walking. | Apple Watch |
| Heart rate average | Overall average heart rate. | Apple Watch |
| Max heart rate | Maximum heart rate. | Apple Watch |
| Heart rate variability | Heart rate variability. | Apple Watch |
| Walking speed | Average walking speed (requires iOS14 or later). | iPhone and Apple Watch |
| Step length | Average step length. Mobility metric (requires iOS14 or later). | iPhone and Apple Watch |
| Walking asymmetry | Proportion of steps which are asymmetrical (0 to 1 range) (requires iOS14 or later). | iPhone and Apple Watch |
| Walking double support | Proportion of time spend with both feet on the ground when walking (0 to 1 range) (requires iOS14 or later). | iPhone and Apple Watch |
| Stair ascent speed | Average speed ascending stairs. | iPhone and Apple Watch |
| Stair descent speed | Average speed descending stairs. | iPhone and Apple Watch |
| Six-minute Walk | Distance walked in 6 minutes. | iPhone and Apple Watch |
| Walking steadiness | Walking steadiness (0 to 1 range) (requires iOS15 or later). | iPhone and Apple Watch |
| Average environmental noise exposure | Average environmental audio exposure (requires Apple Watch Series 4 or later). | Apple Watch |
| Max environmental noise exposure | Maximum noise level in environment. | Apple Watch |
| Average headphone noise exposure | Average audio exposure through headphones. | iPhone |
| Max headphone noise exposure | Maximum audio exposure through headphones. | iPhone |
